# Surgical Sketches

**Published:** 1853-02

**Authors:** W. E. Horner

**Affiliations:** Prof. of Anatomy in the University of Pennsylvania, Senior Surgeon at the St. Joseph’s Hospital, &c. &c.


### MEDICAL EXAMINER,

AND

### RECORD OF MEDICAL SCIENCE.

NEW SERIES.—NO. XCVIII.—FEBRUARY, 1853.

---

### ORIGINAL COMMUNICATIONS.

*Surgical Sketches.* By W. E. Horner, M. D., Prof. of Anatomy in the University of Pennsylvania, Senior Surgeon at the St. Joseph's Hospital, &c. &c.

*A Military Hospital at Buffalo, New York, in the year* 1814.

(Continued.)

In regard to wounds from grape and cannister shot, they are more torn, owing to the larger size of such projectiles. And from the greater momentum much more mischief is done in the laceration of the soft parts, and in the comminution of bones, than happens from musket balls. Such missiles pass straight on, and, except when the surface of the body or the limbs are concerned, they are for the most part immediately fatal, or in a short time. Musket balls, on the contrary, have their course very much varied by the material resistance they meet with in the different tissues and especially by the angle at which they strike. Hence it is not unusual for them to pass around a part one half of its circumference immediately under the skin, giving delusively the appearance of a wound through and through.

If the smaller spherical projectiles from cannon make terrible wounds, such of the latter as arise from the angular fragments of exploded hollow shot are still more formidable in the rent and destruction of parts, and there are still fewer recoveries from them. Rail-way accidents and steam explosions produce a class of accidents very nearly allied to them.

I have said that in the lighter wounds, poultices were early dispensed with; the inflammatory symptoms were mild, and subsided as the discharge of pus increased. The local treatment in common use, was the daily washing of the wounds with soap and warm water, and a simple plaster of basilicon spread on patent lint. We were, however, frequently in want of the latter, and its place was tolerably well supplied by slips of the cotton muslin which we used for bandages. When the sores showed an indisposition to heal, they were washed freely with French brandy or with whiskey. The poultice of bread and milk, of flaxseed, or of slippery elm, was resorted to with great advantage in soothing the irritability and extreme sensibility of some wounds. Situated, however, as we were, milk was a scarce article, and the bread of an inferior quality. The cold water dressing, since so much eulogized, was not attempted except in the form of saturnine lotions. Having been in Paris in the celebrated outbreak there in June 1848, and witnessed the cold water dressings in several of the Hospitals, I can not say that I considered it equal in comfort and in efficacy in severe cases to the preceding well established routine of warm poultices. It may retard inflammation and also repress it, and in these two lights may do well; but another important indication in many wounds is to make provision for the tumefaction which follows them, and nothing relaxes rigid tissues with more certainty than hot fomentations and poultices. Any one who will try the experiment of treating a sprained ankle by cold applications alone, in contrast with large hot poultices, using in both cases leeching besides, will be convinced of the vastly superior efficacy of the poultice, and see that the treatment is abridged nearly one half in point of time by the use of the latter. On the occasion alluded to in Paris, it afforded me pleasure to perceive that the dressing of wounds in the wards of the Hotel Dieu, and of the Saint Louis, was still by the warm well made poultices, while the cold water fomentation or irrigation was almost exclusive in the Val de Grace, (a military hospital.) In the Neckar, the treatment was eclectic, some patients being dressed with cerate, spread on perforated linen, others with poultices, and others with cold water.

I have in a preceding place, alluded to the paramount interest attaching to amputations, and especially the period at which they should be performed. This question was for a long time considered as settled upon the authority of the leading surgeons of the French and of the British Armies, during the great wars at the beginning of the present century. They generally asserted their conviction in favor of amputation directly after an injury, instead of waiting for two, three or more weeks. The ancient doubts are now, however, beginning to revive, and the more recent statistics are received with no small attention.

The frequency of fatal results upon direct amputation leads to the inquiry necessarily, whether a severe operation upon the heels of a severe accident is not, however well performed, more injurious than beneficial to a patient, and whether, in view of preventing an ulterior harm, it does not produce an immediate mischief? Whether the true course in such cases would not be either to let the limb undergo the ordinary treatment of poultices, &c., or if the nourishment of it could not be kept up beyond the injured spot, to do no more than simply cut it off through the ray of connexion which it may have with the part above. In reproducing such ideas it is to be admitted that circumstances are to regulate, in a measure, the proceeding. When the patient can be placed at once in convenient quarters, the refraining from amputation has much to recommend it. But when he has to be transported roughly a great distance, the amputation of the thigh, or at least the immediate excision of the dangling part of the member, would probably be better than to leave all as the accident placed it. It appears to me that the leading inducement to many amputations immediately after injury is only the making of a better stump; but nature has great capacity in this respect, in the most unseemly injuries; and by her law if a bone protrudes beyond its limit of covering by muscle and skin, she in a few weeks, reduces its length to the proper mark by the process of exfoliation. This I have frequently seen accomplished when a spontaneous retraction of the muscles from the bone had denuded it.

Wiseman, surgeon to Charles II. of England, counselled that if there be no hopes of saving the member, the amputation should be done upon the receipt of the wound, before the patient's spirits were overheated with pain or fever, his strength impaired by loss of sleep, and while he is still amazed, as it were, with the accident. Le Dran was of the same opinion, and especially in the case of wounded joints. Ranby, surgeon to George II. in the fore part of the last century, deriving his experience from the campaigns in Flanders, advised amputation on the spot, even on the field of battle. But the most prominent authority of the last century is Bilguers, Surgeon General to the Prussian Army under the great Frederick, who in 1762* published against amputation in general. He denied its necessity even when a limb was torn off by a cannon ball and the parts were hanging. With these views he permitted no amputations in the Prussian Army. The exposition of his success is that, having at one time 6618 wounded patients in hospital, 5557 were perfectly cured, 195 almost restored, 213 remained incapable of either military or civil labor, and 653 died. The 195 and 213 had their bones broken, that is 408, and of the 653 deaths, 408 were from fractured bones. There stand 408 saved without amputation, and 408 deaths also without it. He then says that if we compare the number saved without amputation, with the almost total loss of patients after amputation (only one or two escaping) out of a prodigious number operated on in the beginning of the war, we may safely conclude that the 408 saved would have been victims to the operation. Exceptions are taken to this conclusion by Mr. Guthrie, because Bilguer makes no account of the wounded who died upon the field of battle for the want of amputation. There is, however, such an immense difference between only one or two saved in the beginning of the war, and 408 saved under a new regulation, that if Bilguer speaks candidly and correctly the assertion is of great weight.

About the middle of the past century, (1756) the French Academy of Surgery made this subject a prize question, which was decided in favor of M. Faure, a military surgeon of experience. He adduced ten cases of experiment on delayed amputation, following the battle of Fontenoy in 1745, all successful, while his opponents brought forward only four cures, out of nine immediate amputations. Faure's eligible period for amputation

* See Guthrie on Gun-shot wounds.

was when the violence of the local inflammatory condition and the symptomatic fever had abated. He, however, permitted prompt amputation in some few cases, they being of the worst kind, and not admitting of any delay whatever. Faure informs us that of three hundred amputations upon the battle of Fontenoy, only thirty were successful.*

In 1792 this decision of the French Academy was reiterated by the Baron Percy in his Manuel du Chirurgien d'Armée. The celebrated John Hunter, says on the subject,† after excepting cases where there is danger of death from hemorrhage, and the blood-vessel can not be reached without amputation, that it is much better to wait till the inflammation, and all its effects with those of irritation be gone. And this caution he applies with marked emphasis to the lower extremity. But few, he says, can support the loss of a lower extremity when they are in full health and vigor. "We know that a violent inflammation will in a few hours alter the healthy disposition, and give a turn to the constitution, especially if a considerable quantity of blood has been lost, which will most probably be the case when both accident and operation immediately succeed one another." He reprobates decidedly amputation on the field of battle,‡ the motive for which must of course be to avert the inflammation of the part which would follow its neglect; but he says, that if the patient would be able to sustain the inflammation, a consequence of the accident alone, it is more than probable he would not be able to support the amputation and its consequences too. If chances are so even for and against the patient when amputation is performed under ordinary circumstances in life, how adverse they become upon a field of battle." We must confess that we consider these pregnant precepts in regard to military surgery, but too applicable to the more pacific, but no less terrible series of injuries resulting from railroads, the actual extension of which at present in our country, and their still further extension for the future, urges imperiously a sound rule for the guidance of every practitioner.

* See Larrey, Memoirs of Milit. Surg, &c., Balt. Edit. 1814. Vol. 2, p. 79.

† A Treatise on the Blood &c., p. 537, Phila. Edit. 1840,

‡ Ib. p. 538.

In an army, additional ease in travelling, and especially in a hostile country may be a motive for prompt amputation, but this can have only a limited influence in civil practice, and therefore ceases to be an argument. Mr. Hunter proceeds to say that few did well who had their limbs removed on the field of battle, while a much greater proportion have recovered upon amputation after the inflammation was over. He is, however, less scrupulous about prompt amputation of the upper extremity, or in any case when the limb holds by only a small connexion. My own doubts, I admit, extend even to the latter, and I sincerely desire that a proper test should be applied by experiments of a suitable kind. It can do but little harm when a limb is dangling, and with the bones comminuted, to resect the limb at the point of connexion, and then simply to square off the protuberant bone. By this forbearance, which is scarcely an amputation, we avoid a new invasion of parts already in a suffering state.

The introduction in the year 1782, by Mr. Alanson, of the plan of treating amputated limbs by procuring an immediate union of the flap with the stump, may be considered as giving the strongest impulse to the idea of prompt amputation after injury; it is said to have diminished considerably the mortality when compared with that of the preceding forty years. This is, however, a pure question of comparative treatment of amputation, in which the English and the French schools are still opposed. The intelligence and ardor of the latter do not admit of a doubt that they are governed in their own practice by the results or the convictions of experience, and not by a simple routine as is alleged by their insular Neighbors. But this question does not reach the fundamental one of the relative superiority of a prompt, or of a tardy, or of a delayed or remote operation, the great one for the present stage of civil surgery.

The instructed reader need scarcely be informed that English surgery has run exclusively into the practice of prompt amputation after an accident likely to produce final loss of limb; that the celebrated Baron Larrey has used his high influence in producing a similar practice in France; and that these two sources of authority have established the principle in American surgery. The opinions of our army surgeons in the war of

1812 all went that way, so far as we can learn, through the unfortunately too limited publications on that subject; and we presume that these opinions have been reproduced in the late Mexican war, though unhappily there are so few means of ascertaining the case. Notwithstanding this, we are yet constantly admonished by the frequency of death after immediate amputation, that if the practice be a sound one, it is at least very unsatisfactory in its general results.

Mr. Guthrie* makes what he considers a triumphant statement in favor of his own views for immediate amputation; to-wit: in 163 amputations of the upper extremities, five only died, the rest were cured or convalescent; of the lower extremities, 128 amputations, only nineteen deaths, the rest cured and convalescent. His average of success was, in the case of the upper extremities, one death to twelve recoveries, and in the case of the lower extremities, one death to three recoveries. In the delayed amputations in the British Hospitals for the last six months of the year 1813, one hundred and sixteen persons out of 296 died in the case of the upper extremities; and in the case of the lower extremities 149 out of 255, a loss of more than a third in one category, and of much above one-half in the other. By this it appears that nearly one-half of the wounded are lost in the delayed amputations, while not more than a twelfth are lost on the field of battle.

Mr. Guthrie presents a remarkable statement of the mortality attending amputations between the fourth and the eighteenth day, mostly of the thigh. Out of forty-six, forty died. One who has seen amputations at this period of gun-shot wounds, will not forget the horrific yells of the sufferer; and we must confess some surprise at the persistence of a surgeon who would progress through such a scene. His confidence must have been strong in the accuracy of his principles, and his solace is to be found in the statement following, that an equal number, not so badly wounded, but not operated on, also died. This occurred in a group of one hundred and fifty French soldiers, captured at the battle of Salamanca.

* On gun-shot wounds, p. 42. London, 1815.

† Loc cit. p. 59.

Mr. Hennen* is so firmly of the opinion above, that he considers it almost libellous to impute any other practice to English surgeons. The fact of its propriety is, he says, as firmly established as any in surgery, and that there is not one point where opinions have varied so little among English practitioners from Wiseman downwards. The statistics of Mr. Guthrie and the axiom of Mr. Hennen, have thus a strong contrast with the celebrated Prussian surgeon Bilguers, and with the deeply reflective mind of John Hunter. It is said that the Indians of the Rocky Mountains consider a few perforations with rifle balls or penetrations with arrows, as not a very serious matter; that they suffer comparatively but little inconvenience from such accidents. If vital parts are not struck they do not lay up, but let these missiles drop out pretty much as they can. All of which is stated to be due to the singular salubrity of the air. The air of a field of battle appears to have a similar protective influence upon the constitution of the British soldier, or else there are some qualifications in the condition of things which statistical tables fail to represent. We can scarcely doubt the sincerity of Mr. Guthrie's views, whatever may be the striking character of his reports. He certainly had great opportunities for experience when war was conducted on so extensive a scale. But whatever may be his convictions in regard to early amputation, he is decided in allowing the first moments of agitation to pass before the operation is performed. He admits a period of from one to six or eight hours in different individuals, but considers that from one to three hours will, in most cases, be sufficient.†

The great battle of Waterloo, fought on the 16th and 18th of June, 1814, left two thousand killed in the British lines, and eight thousand wounded; the number of amputations amounted to nearly five hundred, in more than one-third of whom the operations had been prompt. We are informed by a careful observer, who made his report on the state of the British Hospitals‡ in Belgium at the time, that the mortality among those where am-

* Observations, &c., on Military Surgery. Edinburgh, 1818, pp. 45, et seq.

† Loc. cit., p. 52.

‡ John Thompson, Regius Professor, &c. Edinburgh, 1816.

putation was not at all performed, and among those where it had been postponed, was so much in excess over the cases of mortality after the cases of prompt amputation, that many regrets existed among the army surgeons that primary amputations had not been more frequently performed. This axiom then of British practice, established on the most memorable occasion of modern times, has met with but little to disturb its ascendancy since, and may now, therefore, be viewed as the dominant doctrine of the profession there.

In a recent lecture by Mr. Guthrie,* his opinions previously expressed on the result of his Peninsular service, are reiterated in substance as follows, to-wit: Immediate resection when the limb is in a hopeless state. The exception being when the patient is so prostrated as to render it evidently hazardous at once to his life. A delay of from five to eight hours and the use of stimulants are then recommended. He considers amputations done within the first twenty hours, over such as are done after several days or weeks, so superior as to admit no longer of dispute. He appears, however, to omit the question whether amputations at any period have an advantage over no amputations at all. He admits that amputations below the shoulder joint downwards, and below the knee downwards, may almost always be done with safety, but the sooner the more sure. Amputations any where above the middle of the thigh have always considerable danger. The latter being, then, the really turning point of the enquiry, the question should first of all be settled, whether immediate amputation, delayed amputation, or no amputation at all of this part, be better; and as immediate or delayed amputation in the two other cases do not differ materially in their results, so incidental circumstances may direct the surgeon. But it may be doubted always, I say, whether the immediate repetition to the limb of a severe injury by amputation is likely to be so balmy, as the admission of a fair interval of time. I consider it highly inconclusive to group amputations by the limb instead of by the region of the limb, and still more so to speak of all sorts of amputations in a sum, without any analysis.

* See American Medical Journal, Oct. 1852, p. 530, from Lancet, May 1, 1852.

The surgeon most influential in obtaining the present professional conviction in favor of immediate amputation after injuries requiring the resection of the limb, is unquestionably the celebrated Baron Larrey.* His surgical life began in 1789, and was continued with great activity for the next twenty-six years, when the downfall of the Emperor Napoleon composed the disturbed state of Europe. In this long service he was the constant attendant of his celebrated master, and present at all his great battles as Surgeon-in-Chief. His amputations were sometimes fifty or sixty a day, and, on one occasion, amounted to two hundred. Seeing for himself to such an amazing extent, and receiving the most authentic reports from all quarters, it would be difficult to find elsewhere such an amount of information. It would appear that under his observation more than three-fourths have recovered, which he ascribes to a more correct appreciation of the time for operating, to more methodical dressing, and to a more simple and less painful process than in preceding times. His rule is, that when a limb is so shattered that it cannot be saved, amputation should be done in the first twenty-four hours.†

Immediate amputation is, however, far from being the universal doctrine of the French schools. The Baron Percy, as previously remarked, disallowed it in his manual for army surgeons, published, in the year 1792, but understood to have been well received at a comparatively recent date. And Blandin, so late as 1829,‡ leaves us with the same conclusions in regard to his sentiments.

The American surgeons, in the war of 1812, followed to a large extent the prevailing French and English practice of that period ; among them we may mention Dr. Mann ; yet he states that after the battle of Little York and Fort George, a less number survived primitive than consecutive amputation. Three or four, it appears, died immediately after the operation, whereas there was not a single case of death during the campaign (1813) occasioned by consecutive amputation § We regret that he has not furnished us with the results of more than thirty amputa-

* See Memoirs of Milit. Surg., passim. Balt. Ed. 1814. Translation by R. W. Hall, M. D.

† Loc. cit. p. 79.

‡ See Dict. de Med. et de Chirurg. Prat. Art. Amputation.

§ Medical Sketches, p. 213.

tions executed after the battle of the 11th of September, 1814, on Lake Champlain, rendered more memorable by the naval victory of Commodore McDonough. Dr. Amasa Trowbridge,* also an experienced surgeon of that time, and still alive, has given his testimony in favor of immediate amputation when there is no prospect of the ultimate restoration of the limb.†

In a valuable communication under date of April 30, 1851, from Dr. J. B. Whitridge, now of Charleston, S. C., and one of the U. S. Army surgeons of the war of 1812, he has furnished me with his views generally on the subject of amputation. Among other remarks he says: "According to my observations and experience amputation should always be performed as soon as possible after the accident or wound has taken place, which creates the necessity of the operation." He enjoys the reputation of a highly skilful and successful surgeon.

In the Naval fight on Lake Erie, September 10th, 1813, Dr. Usher Parsons,‡ the fleet surgeon, adopted the same plan of immediate amputation. The American squadron contained about six hundred, all told. The flag ship, the Lawrence, under Commodore Perry, had mustered in the morning one hundred men fit for duty. The action lasted three hours, and left at 3 o'clock P. M., twenty-one dead and sixty-three wounded, only sixteen unhurt. The whole number of wounded in the squadron

* This gentleman, though now at an advanced age, exhibits an unflinching energy in the exercise of his profession. His residence being in Watertown, in the northern part of the State of New York, he has enjoyed for nearly half a century a most prominent reputation as an operator. In recent communications from him, he reports ninety amputations of the thigh in private practice since 1809. Of the first thirty-five, only one died immediately after the operation; the remainder recovered from the operation, and continued to live for a well marked time afterwards. His report in detail was published in the Boston Medical and Surgical Journal. He lately performed his eighteenth operation (lithotomy) for stone in the bladder, this case terminating, as all the previous ones, in success. Of these, three were instances of foreign body in the bladder, one being a slate-pencil two inches long, one a piece of willow stick one and a half inches long, and one a bit of sealing wax two inches long and three-fourths of an inch in circumference. In thirteen cases of tracheotomy for foreign bodies, twelve were successful.

† See Boston Med. and Surg. Journ., 1838.

‡ See New England Journal of Med. and Surg., Oct. 1818, p. 313.

was ninety-six, of these, only three died; one from compound fracture of the shoulder, in which the bones were in part carried away, one from mortification of the lower extremity, and a third from fracture of the skull. The moral influence of victory was strongly illustrated in this engagement. It is known that the flag ship struck her colors; medical aid, the Doctor tells us, was then rejected, and the cry at once was, "sink the ship, let us all sink together." The translation of the Commodore to the Niagara, and the bringing of that ship into action, changed the fortune of the day, and shouts of victory immediately ascended. Its influence was seen in the remarkably few deaths of the wounded. Dr. Mann says that the same happy consequences attended the victory on Lake Champlain. The prodigious and glorious successes of the British arms in the Peninsular campaigns may account for many of the recoveries there also.

### APPENDIX.

The following tabular statements on this subject may be presented with some advantage. In the Pennsylvania Hospital it appears from the report of Dr. George W. Norris, one of the surgeons,* that there had been fifty-six amputations from January 1, 1831, to January 1, 1838, a period of seven years, under the following circumstances:

*Pennsylvania Hospital.*

| | Aggregate Amputations. | Died. | Recovered. | Accidental Amputations, or prompt. | Died. | Recovered. | Amputation delayed or from disease. | Died. | Recovered. | Total. |
|---|---|---|---|---|---|---|---|---|---|---|
| Thigh . . . | XIII. | 6 | 7 | VI. | 4 | 2 | VII. | 2 | 5 | 13 |
| Leg . . . | XVI. | 9 | 7 | IV. | 1 | 3 | XII. | 8 | 4 | 16 |
| Foot . . . | IV. | 1 | 3 | I. | 1 | | III.‡ | | 3 | 4 |
| Shoulder Joint . | II. | 2 | | I. | 1 | | I. | 1 | | 2 |
| Arm . . . | VI. | 2 | 4 | IV. | 2 | | II. | | 2 | 6 |
| Fore arm . . | XIII. | 2 | 11 | VIII. | 2† | 6 | V. | | 5 | 13 |
| Hand . . . | II. | | 2 | | | | II. | | 2 | 2 |
| | | 22 | 34 | | 11 | 11 | | 11 | 21 | 56 |

*See Amer. Jour. Med. Sci. vol. 22, p. 356.

† Dr. Norris has not stated whether these were after prompt or delayed amputation.

‡ Two on same patient.

*Pennsylvania Hospital from Jan.* 1, 1838, *to Jan.* 1,1840.*

| | Aggregate. | Died. | Recovered. | Prompt. | Died. | Recovered. | Delayed. | Died. | Recovered. | Total. |
|---|---|---|---|---|---|---|---|---|---|---|
| Thigh . . . | IV. | | 4 | | | | IV. | | 4 | 4 |
| Leg . . . | X. | | 10 | VII. | | 7 | III. | | 3 | 10 |
| Foot, partial . . | I. | 1 | | I. | 1 | | | | | 1 |
| Arm . . . | VI. | | 6 | III. | | 3 | III. | | 3 | 6 |
| Fore arm . . | III. | | 3 | I. | | 1 | II. | | 2 | 3 |
| | | 1 | 23 | | 1 | 11 | | | 12 | 24 |

In regard to the Massachusetts General Hospital, it appears from the Report,* including the amputations from January 1822, to January 1, 1850, that the aggregate had been one hundred and forty-six on one hundred and forty-one patients, thirty-two of which had died:

85 in consequence of disease, of whom 10 died, i. e. 1 in 8½ cases.
56 " injury, " 22 " 1 in 3 "

I have condensed the report into the following table:

| | Aggregate. | Died. | Recovered. | Prompt Amputation. | Died. | Recovered. | Delayed. Amputation. | Died. | Recovered. | Total. |
|---|---|---|---|---|---|---|---|---|---|---|
| Thigh . . . | LXIX. | 19 | 50 | XIV. | 7 | 7 | LV. | 13 | 42 | 69 |
| Leg . . . | L. | 10 | 40 | XIII. | 3 | 10 | XXXII | 6 | 31 | 50 |
| Arm . . . | XI. | 1 | 10 | III. | 1 | 2 | IX. | | 9 | 11 |
| Fore arm . . | XI. | 2 | 9 | V. | 1 | 4 | VI. | | 6 | 11 |
| | | 32 | 109 | | 12 | 23 | | 19 | 87 | 141 |

* See Amer. Journ. Med. Sc., vol. 26, p. 36. Paper by Dr. George W. Norris.

† By George Hayward, M. D., one of the surgeons. See Boston Med. and Sur. Journal, Oct., 1850.

*New York Hospital.**

| | Aggregate Amputations. | Died. | Recovered. | Accidental Amputations, or prompt. | Died. | Recovered. | Amputation delayed, or from disease. | Died. | Recovered. | Total. |
|---|---|---|---|---|---|---|---|---|---|---|
| Thigh . . . | XXXIV. | 11 | 23 | IX. | 3 | 6 | XXV. | 8 | 17 | 34 |
| Knee Joint . . | I. | 1 | | | | | I. | 1 | | 1 |
| Leg . . . | XXIV. | 7 | 17 | XV. | 6 | 9 | IX. | 1 | 8 | 24 |
| Shoulder Joint . | IX. | 4 | 5 | VII. | 4 | 3 | II. | | 2 | 9 |
| Arm . . . | XI. | | | VIII. | | 8 | III. | | 3 | 11 |
| Fore arm . . | XI. | 3 | 8 | VI. | 1 | 5 | V. | 2 | 3 | 11 |
| | | 26 | 53 | | 14 | 31 | | 12 | 33 | 90 |

It is to be regretted, I say, that we have not more statistics of the result of amputations in our Mexican army. Immediate amputation appears to have been the favored practice. "No rule was more universally acted upon by the surgeons of our army in Mexico, from the battle of Palo Alto to the treaty of peace, than the one laid down by Hennen, with as little delay as possible.†

As this was so uniform a proceeding, the opposite had but a very imperfect trial, and the statistics of success not being given by Dr. Porter in his paper, there are no means of comparing with the results in other places. We hope that some gentleman of the army will devote himself to the collecting of facts on this subject while they are still so accessible.

We are informed by Dr. Richard McSherry, of the U. S. Navy,‡ that he did not see in his own practice, or in that of any other surgeon, a single case of cure after severe injury of the thigh or knee, either with or without amputation. This was in an attendance of eight months in the hospitals of the city of Mexico, after the fighting was over, and it applies to wounds fracturing the os femoris. All other wounds seemed to do as well in that city as in other

* See American Journal Med. Sc., July, 1848. Statistics, &c., of Amputations from Jan. 1, 1839, to Jan. 1, 1848.

† Medical and Surgical Notices, &c., by John B. Porter, M. D., surgeon U. S. Army, in American Journal of the Medical Sciences for July, 1852.

‡ See Amer. Jour. Med. Sc., July 1849.

climates, and he presumes that not one case in twenty was fatal after amputation of the arm.

*Report of* Prof. Restelli, *of the Sardinian Army.**

| | Aggregate. | Died. | Recovered. | Prompt Amputations. | Died. | Recovered. | Delayed. | Died. | Recovered. | Total. |
|---|---|---|---|---|---|---|---|---|---|---|
| Hip Joint . . | I. | | 1 | I. | | | | | | 1 |
| Thigh . . . | XVIII. | 10 | 8 | XI. | 4 | 7 | VII. | 6 | 1 | 18 |
| Leg . . . | IV. | 1 | 2 | | | | I. | 1 | | 4 |
| Shoulder Joint . | III. | 1 | 2 | I. | | 1 | II. | 1 | 1 | 3 |
| Arm . . . | XVII. | 6 | 11 | X. | 1 | 9 | VII. | 5 | 2 | 17 |
| Fore arm . . | III. | 1 | 2 | | | | III. | 1 | 2 | 3 |
| | | 19 | 26 | | 5 | 17 | | 14 | 6 | 46 |

The conclusion drawn from the above, by Prof. Restelli is, that prompt amputations are more felicitous in their results, than delayed.

In the African campaigns of the French in 1837, '38, and '39, according to the report of Dr. Guyon, of sixty-three amputations of all the limbs, including six at the shoulder-joint, seventeen patients died and forty-six recovered, and the results were about the same in prompt and in tardy operations. The report is, however, very unsatisfactory, from the want of details in regard to precise time, and to deaths in relation to the places of amputation. For, by putting into one sum all the deaths, it confounds things widely separated in other respects,† and leaves us without guide, at least so far as the objects of the present observations are concerned.

The report of M. Malgaigne in regard to the results of amputation in the Paris hospitals for traumatic lesions, from 1836 to 1846, is a remarkable document.‡ For example in

* See British and Foreign Med. Chirug. Review. Oct., 1850.

† See Brit. and For. Medical Review, vol. xii. 1841. From Gazette Medicale.

‡ See Amer. Journ. Med. Sc. New Series, Oct., 1848, p. 468. From Med. Times.

| | | | |
|---|---|---|---|
| Thigh, | 44 amputations. | 34 deaths, | over 3 in 4 |
| Leg, | 67 " | 42 " | nearly 2 in 3 |
| Foot, | 8 " | 5 " | over 1 in 2 |
| Shoulder, | 7 " | 7 " | Total fatality. |
| Arm, | 29 " | 17 " | nearly 2 in 3 |
| Fore arm, | 10 " | 2 " | 1 in 5 |

The events of 1848, when a large number of wounded insurgents were admitted into the St. Louis Hospital, appear to have left M. Malgaigne and his colleague, M. Gosselin, in the same discouragement on the subject of amputations being performed at all; and it seems that he has reached the conclusion that the opinion of military surgeons on the advantage of primary amputations, did not rest upon a very solid basis, and that in the attempt to preserve the limbs of the wounded, the surgeon did not place them in greater hazard than in amputation. It thus happens that after half a century or more of established opinion on this great point, it is now in a state of vacillation, and may possibly return to where Bilguer left it in 1762. If there be any thing intermediate to these two extremes, in its application to the present state of civil surgery in regard to railroad accidents, &c., it is, I repeat, to leave the limb, when it is hopelessly injured, without any other resection, than that of cutting off the dangling part, squaring with a saw the end of the bone, and trusting the rest to nature. In our present limited experience on this subject, I can not say that the proceeding is absolutely recommended; but there are arguments enough to invite a fair trial, and especially in compound fractures of the thigh and of the leg, where danger is so imminent, either in amputating or in trusting to nature entirely.

In conclusion, we would recommend that in the tabular form of hospital reports on the subject of amputation, the time should be regularly marked, whether it be under or above four days. Four days is probably the extremity of time for primary or prompt amputation; after that time nothing should be done for fourteen or twenty days more. Then the time for an amputation ranges for the remainder of the disease. Also, no sound reasoning can be founded upon the aggregation into one sum of all amputations, inasmuch as they vary so much in fatality, depending upon the limb. The thigh, the shoulder and the leg, being the most fatal, should each have its respective consideration. The European reports that I have seen are very defective in these respects, and many of them, which would otherwise have been of value to the American reader, are, for the want of such information, passed over on the present occasion. It would be a great acquisition to the profession generally, if some one there near the seat of information, would go to work and reconstruct all of their statistical tables upon a more exact plan. To say that a patient died after amputation, is to say only one half, there may have been circumstances entirely independent of the original injury and of the amputation which led to death. These circumstances may have been of so serious a character as to destroy, of themselves, the patient; as for example—internal injuries besides external; or internal fatal diseases so far advanced that nothing could cure them, as tubercles of the lungs, brought on by caries of bones or diseases of joints. The *post hoc* is always a different consideration from the *propter hoc*, and in no one affair more than in amputations as a class of surgical affections. Our own general conclusion from what we have seen and learned would be, that amputations in the length of the bones of the upper extremity, anywhere below the shoulder joint, may be performed indiscriminately, either at once or subsequently. Perforations by balls through the elbow and wrist, in their ulterior consequences, involve great hazard to the life of the patient; but some patients recover. It is, perhaps, therefore, better to delay amputation, as there is no immediate danger for the most part. Compound fracture of the thigh is imminently dangerous, either with or without regular amputation. An intermediate plan has therefore been suggested, which, for similar reasons, may be applicable to the leg also, to-wit: the excision of the limb through the ray of attachment, and the simple squaring off of the ragged end of the bone.

